# Use of microbiological and patient data for choice of empirical antibiotic therapy in acute cholangitis

**DOI:** 10.1186/s12876-020-01201-6

**Published:** 2020-03-12

**Authors:** Tassilo Kruis, Sarah Güse-Jaschuck, Britta Siegmund, Thomas Adam, Hans-Jörg Epple

**Affiliations:** 1 Labor Berlin Charité Vivantes GmbH, Mikrobiologie & Hygiene, Berlin, Germany; 2 Charité – Universitätsmedizin Berlin, Medizinische Klinik für Gastroenterologie, Infektiologie und Rheumatologie, Campus Benjamin Franklin, Berlin, Germany

**Keywords:** Acute cholangitis, Biliary tract infection, Gastrointestinal tract microbiology, Antimicrobial resistance, Empirical antibiotic therapy, Carbapenem-sparing therapy

## Abstract

**Background:**

Ineffective antibiotic therapy increases mortality of acute cholangitis. The choice of antibiotics should reflect local resistance patterns and avoid the overuse of broad-spectrum agents. In this study, we analysed how results of bile and blood cultures and patient data can be used for selection of empirical antibiotic therapy in acute cholangits.

**Methods:**

Pathogen frequencies and susceptibility rates were determined in 423 positive bile duct cultures and 197 corresponding blood cultures obtained from 348 consecutive patients with acute cholangitis. Patient data were retrieved from the medical records. Associations of patient and microbiological data were assessed using the Chi-2 test and multivariate binary logistic regression.

**Results:**

In bile cultures, enterobacterales and enterococci were isolated with equal frequencies of approximately 30% whereas in blood cultures, enterobacterales predominated (56% compared to 21% enterococci). Antibiotic resistance rates of enterobacterales were > 20% for fluorochinolones, cephalosporines and acylureidopenicillins but not for carbapenems (< 2%). The efficacy of empirical therapy was poor with a coverage of bacterial bile and blood culture isolates in 51 and 69%, respectively. By multivariate analysis, predictors for pathogen species, antibiotic susceptibility and expected antibiotic coverage were identified.

**Conclusions:**

In unselected patients treated for acute cholangitis in a large tertiary refferential center, use of carbapenems seems necessary to achieve a high antibiotic coverage. However, by analysis of patient and microbiological data, subgroups for highly effective carbapenem-sparing therapy can be defined. For patients with community-acquired cholangitis without biliary prosthesis who do not need intensive care, piperacillin/tazobactam represents a regimen with an expected excellent antibiotic coverage.

## Background

Acute cholangitis is a potentially life-threatening infection of the biliary tract. The main underlying pathological process is obstruction of bile ducts leading to cholestasis and impeded clearance of intestinal microorganisms ascending from the duodenum. The increase in biliary tract pressure and the local inflammatory response to the infecting organisms impair the integrity of the biliary epithelium, facilitate bacterial translocation into the circulation, and promote systemic inflammation [1]. In addition, certain procedures, such as sphincterotomy, stent placement or biliodigestive anastomosis, can predispose to bacteriobilia and cholangitis. In these situations, infection of the biliary tract may develop in the absence of obstruction [1, 2].

The clinical course of acute cholangitis ranges from mild abdominal symptoms to sepsis and shock. Despite considerable progress in the treatment of acute cholangitis during the last decades, recent studies still report mortality rates of up to 10% [2, 3]. As with other localized infections, treatment success depends on efficient source control and appropriate antibiotic therapy. Source control is achieved by biliary decompression by endoscopic or percutaneous drainage. The importance of antibiotic therapy is highlighted by the fact that inappropriate empirical therapy is associated with increased mortality [3].

Studies from different geographic regions of the world consistently found *E.coli, Klebsiella spp.* and *Enterococcus spp.* as most common pathogens in ascending cholangitis [4]. Thus, the microbial spectrum to be covered by empirical therapy seems to be well defined in general. However, individual patient factors as well as local resistance patterns can increase the probability for infection with resistant enterobacterales, *P.aeruginosa* or other difficult-to-treat pathogens [5]. Enterobacterales resistant to third generation cephalosporines (3GCRE) are of particular importance because their treatment regularly requires the use of carbapenems. Because of the rising prevalence of cephalosporine resistance, the clinician is confronted with the dilemma of ensuring broad empirical antibiotic coverage while at the same time avoiding excessive use of carbapenems. Particularly in institutions with a high prevalence of 3GCRE, it seems difficult to define a role for carbapenem-sparing regimens. Knowledge of local susceptibility rates alone is not sufficient to solve this problem.

In our study, we investigated whether the analysis of patient factors and their association with pathogens and antibiotic resistance could be helpful to avoid overuse of carbapenems and other broad-spectrum antibiotics. To this end, we conducted a retrospective analysis of 348 patients treated for ascending cholangitis in Germany’s largest university medical center. We determined the pathogen spectrum and antibiotic susceptibilities of enterobacterales and enterococci in biliary and blood samples from these patients. Particularly, we analysed the significance of various patient factors for predicting the isolation of bacterial biliary pathogens and their antibiotic susceptibility profile. Our results demonstrate, how the combination of microbiological and patient data can be used for the choice of efficacious antibiotic therapy of patients with acute cholangitis.

## Methods

All consecutive specimens sent to the microbiological laboratory of the Charité (Institute of Microbiology and Hygiene of the Charité until December 2010; Labor Berlin Charité Vivantes GmbH, thereafter) from January 2007 until July 2015 were screened for positive bile cultures obtained by endoscopic or percutaneous sampling. From all patients with positive bile cultures, blood culture results were also extracted, if they had been collected within 48 h from the corresponding bile sample.

**Fig. 1:**
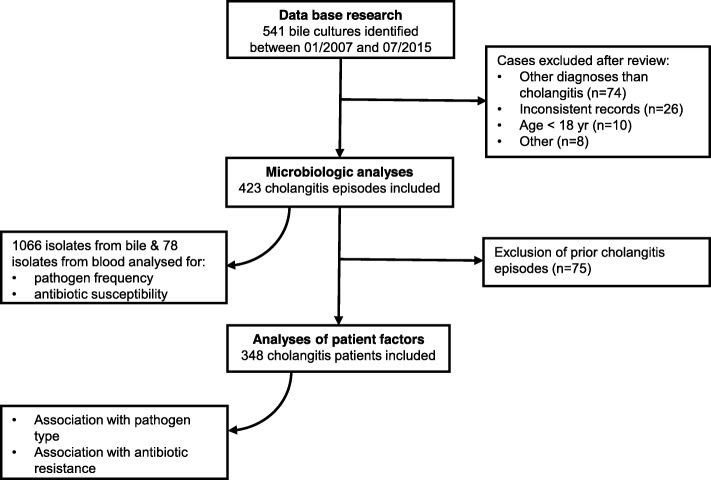
Study design

The diagnosis of cholangitis was reviewed against the patient records. Bile cultures that were not collected within a course of cholangitis were excluded. Samples were also excluded, if the patient’s age was < 18 years or clinical data were incomplete or inconsistent. Figure 1 depicts the schematic structure of the study.

Bile specimens were cultivated on conventional solid media and incubated in aerobic and anaerobic atmospheres at 37 °C. Aerobic microorganisms were routinely identified and tested for antibiotic susceptibility using the VITEK 2 System (bioMérieux, France). Antibiotic susceptibilities were interpreted according to EUCAST recommendations (http://www.eucast.org/). If necessary, additional commercial methods were applied such as matrix-associated laser desorption/ionization time-of-flight mass spectrometry (MALDI-MS; Bruker, Massachusetts, USA, before 2011, or bioMérieux, France, since 2011) for species identification or the disk diffusion method and E-tests for antibiotic susceptibility testing. Anaerobes were identified by MALDI-MS and tested for susceptibility using ATB ANA (bioMérieux, France) or E-tests. For this study, “intermediate” (I) results in antimicrobial sensitivity testing (AST) were considered “resistant” (R). For enterobacterales, AST was done using ampicillin/sulbactam, piperacillin/tazobactam, cefotaxime, meropenem, and ciprofloxacin. Enterococcus strains were investigated on ampicillin and vancomycin resistance, respectively. Blood cultures with Coagulase-negative Staphylococci (CoNS) in only one pair of bottles were considered contaminated. In line with the recommendations of the German Commission for Hospital Hygiene and Infection Prevention at the Robert Koch Institute (KRINKO) [6] enterobacterales, *P. aeruginosa*, and *A. baumannii* complex isolates were classified as multi-resistant gram-negative rods (MRGN) if they were resistant to three or more of the following substance classes: acylureidopenicillins, third generation cephalosporins, carbapenems, fluorochinolones.

Patient data were extracted from the hospital records. In patients hospitalized several times for recurrent cholangitis, only the most recent episode was analysed. Also, in patients with more than one procedure of bile sampling during the same hospital stay, only culture results obtained during the first sampling procedure were considered for correlation of patient factors with microbiological data. Data on empirical antibiotic treatment were available for 229 patients.

The statistical evaluation was carried out after consultation with the Institute for Biometry and Clinical Epidemiology of the Charité - Universitätsmedizin Berlin using SPSS version 23.0. Significant differences in categorical variables were assessed using the Chi-2 test. Predictors with *p* ≤ 0.2 in the univariate analysis were then tested using multivariate binary logistic regression through backward elimination and forward selection. The significance level in two-sided testing was *p* < 0.05.

### Ethics, consent and permissions

The study was approved by the internal review board (local ethics committee of Charité – Universitätmedizin Berlin; registry number EA2/033/16). The need for informed consent was waived for this retrospective study.

## Results

### Microbiological spectrum and antibiotic susceptibilities

Overall, 541 bile cultures sampled within the study period were identified. After checking for inclusion and exclusion criteria, 118 cases were excluded (Fig. 1). From the remaining 423 bile duct cultures, a total of 1066 microorganisms (bacteria or fungi) were isolated with polymicrobial growth detected in 69% of cultures. The most frequent pathogens isolated were enterococci (31%) and enterobacterales (30%), followed by *Candida spp.* (17%), streptococci (7%), and anaerobes (5%). *P. aeruginosa* represented 2% of bile culture isolates (Table 1). For the purpose of this study, only bacterial pathogens were considered for further evaluation.

**Table 1:** Frequency of microorganisms isolated in bile and blood

	**Bile**	**Blood**
	*(n = 1066)*	*(n = 78)*
***Enteroccus spp.***	31.0%	20.5%
**Enterobacterales**	29.5%	56.4%
*E.coli*	13.2%	32.1%
*Klebsiella spp.*	8.0%	14.1%
*Enterobacter spp.*	2.9%	5.1%
*Citrobacter spp.*	2.4%	2.6%
Other enterobacterales	3.0%	2.6%
***Candida spp.***	16.9%	3.8%
***Streptococcus spp.***	6.6%	1.3%
**Anaerobes**	4.9%	1.3%
**CoNS**	3.3%	3.8%
***P. aeruginosa***	2.3%	7.7%
**Other**	5.5%	6.4%

n, number of isolates from bile and blood, respectively

CoNS, Coagulase-negative staphylococci

We identified 197 blood cultures collected within 48 h of the respective bile samples. Seventy of these had turned positive. After exclusion of CoNS contaminants (8/70), the rate of positive blood cultures was 32% (62/197). Polymicrobial growth was less frequent in blood than in bile cultures (16% versus 69%, respectively). Complete or partial pathogen concordance with corresponding bile cultures was present in 65% (40/62). While in bile cultures entorobacterales and enterococci were isolated with similar frequencies (approximately 30%), enterobacterales comprised the majority of isolates in blood cultures (56% compared to 21% enterococci). In addition, *P.aeruginosa* was isolated more frequently in blood cultures compared to bile cultures (8% vs. 2%) (Table 1). With the exception of carbapenems, a high level of resistance against all classes of antibiotics tested was found in enterobacterales isolated from bile or blood. Also, at least one third of enterococci isolated from bile and blood were resistant to ampicillin (Table 2).

**Table 2:** Antibiotic resistance of bile and blood culture isolates

	**Bile**		**Blood**	
		*(n/N)*		*(n/N)*
***Enterobacterales***				
Ampicillin/sulbactam	54.2%	167/308	56.8%	25/44
Piperacillin/tazobactam	34.0%	103/303	25.0%	11/44
Cefotaxime	26.9%	83/308	11.4%	5/44
Meropenem	1.9%	6/310	2.3%	1/44
Ciprofloxacin	20.0%	62/310	29.5%	13/44
***Enterococcus spp.***				
Ampicillin	33.1%	103/311	37.5%	6/16
Vancomycin	13.2%	40/302	18.8%	3/16

n/N, resistant isolates/tested isolates

### Patient factors

Considering only the most recent and excluding previous cholangitis episodes, 348 patients with acute cholangitis were included into the analysis of patient factors. As shown in Table 3, patients were characterized by a high burden of comorbidity, a high prevalence of preexisting biliary tract pathologies and a high prevalence of prior biliary tract interventions, reflecting the patient selection expected for a tertiary reference center with associated liver transplant unit. Almost 60% of patients included had an indwelling biliary drainage, almost 30% had a biliodigestive anastomosis, and more than 10% of patients have had liver transplantation (Table 3).

**Table 3:** Patient characterisitics

Total study population	***N*** **= 348**		
		***(n)***	
**Male**	63.8%	222	
	*median*	*min./max.*	
**Age at intervention (years)**	64	18/94	
**Age-adjusted Charlson index**	5	0/12	
Cardiovascular disease	42.2%	147	
Malignoma	40.5%	141	
Liver disease	27.6%	96	
Diabetes	24.1%	84	
Kidney disease	20.4%	71	
Pulmonary disease	15.2%	53	
Neurologic disorder	8.6%	30	
**MDR colonization**	15.6%	59	
**Preexisting biliary tract pathologies**^**a**^	83.0%	289	
Papillotomy	30.7%	107	
Malignant stenosis	30.5%	106	
Biliodigestive anastomosis	28.7%	100	
Choledocholithiasis	23.9%	83	
Benign stenosis	23.6%	82	
Liver transplatation	13.2%	46	
**Prior biliary tract interventions**			
Yes	81.0%	282	
No	18.4%	64	
N.d.	0.6%	2	
	*median*	*min./max.*	
Number of prior interventions^b^	2	0/32	
**Preexisting biliary tract drainage**			
Yes	58.9%	205	
No	41.1%	143	
N.d.	8.0%	28	
**Antibiotic pre-treatment within 90 d**			
Yes	46.6%	162	
No	45.4%	158	
N.d.	8.0%	28	
**Indication for recent intervention**			
Stenosis	25.9%	90	
Choledocholithiasis	21.0%	73	
Catheter related	14.4%	50	
Drainage after LTX	8.0%	28	
Liver abscess	4.9%	17	
Pancreatitis	4.6%	16	
Other	21.3%	74	
**Route of biliary drainage**			
Endoscopic	58.3%	203	
Percutaneous	40.5%	141	
N.d.	1.1%	4	
**Successful biliary decompression**			
Yes	92.8%	323	
No	3.4%	12	
N.d.	3.7%	13	
**Outcome**			
Death	14.4%	50	
Admission to ICU	37.6%	131	
	*median*		
Length of hospital stay (days)	16.5	1/367	

N.d., not determinable/not documented

MDR, multi-drug resistant bacteria including MRSA, MRGN, and VRE

^a^One or more underlying conditions possible in one individual

^b^ The exact number of prior biliary tract interventions was known in 260 cases

Overall, enterobacterales were detected in the bile of 52% (182/348) of patients. In the univariate analysis, enterobacterales were isolated less frequently, if bile sampling was performed later than 48 h after admission, after percutaneous compared to endoscopic sampling, in patients with previous or ongoing ICU treatment (during the current hospital stay), and in patients with biliodigestive anastomosis or transplanted liver (Table 4). Enterococci were detected in 59% (205/348) of patients. They were detected more frequently in patients with a high level of comorbidity (as indicated by a Charlson index ≥5), antibiotic pretreatment, prior biliary tract intervention, indwelling biliary drainage, and biliary stenosis. *P. aeruginosa* was detected in 6% (21/348) of patients. The only risk factor associated with isolation of *P. aeruginosa* in the univariate analysis was ICU treatment.

**Table 4:** Association between patient factors and pathogen frequencies

Univariate analysis												
			***Enterococcus spp.***		**Enterobacterales**		**3GCRE**		**MRGN**		***P. aeruginosa***	
		n		***p*-value**		***p*-value**		***p*-value**		***p*-value**		***p*-value**
**Age**	*< 65y*	180	57.8%	0.664	48.3%	0.134	11.2%	0.621	9.0%	0.592	5.6%	0.823
	*> = 65y*	168	60.1%		56.5%		13.5%		10.8%		6.5%	
**Sex**	*m*	222	58.6%	0.910	53.2%	0.738	15.6%	**0.016**	13.3%	**0.008**	7.2%	0.252
	*f*	126	59.5%		50.8%		6.5%		4.0%		4.0%	
**Charlson Index**	*< 5*	201	53.7%	**0.027**	50.2%	0.384	8.6%	**0.019**	7.5%	0.100	5.0%	0.366
	*> = 5*	146	65.8%		55.5%		17.4%		13.2%		7.5%	
**MDR carrier**	*no*	289	57.8%	0.386	51.6%	0.570	9.1%	**≤0.001**	7.3%	**≤0.001**	5.2%	0.224
	*yes*	59	64.4%		55.9%		28.1%		22.8%		10.2%	
**Time of bile collection from admission**	*≤ 48 h*	153	59.5%	1.000	69.3%	**≤ 0.001**	13.9%	0.509	7.2%	0.147	4.6%	0.488
	*> 48 h*	190	58.9%		38.9%		11.3%		12.3%		6.8%	
**Route of bile collection**	*endoscopic*	203	56.7%	0.438	60.1%	**≤ 0,001**	11.0%	0.402	6.0%	**0.003**	5.4%	0.648
	*percutaneous*	141	61.0%		42.6%		14.5%		15.8%		7.1%	
**ICU**^**a**^	*no*	226	59.7%	0.732	63.3%	**≤ 0.001**	14.0%	0.228	8.9%	0.451	3.1%	**0.002**
	*yes*	122	57.4%		32.0%		9.1%		11.7%		11.5%	
**Antibiotic pre-treatment**^**b**^	*no*	157	52.5%	**0.006**	56.3%	0.372	13.0%	1.000	7.0%	0.179	4.4%	0.345
	*yes*	160	67.9%		51.2%		12.4%		11.9%		7.4%	
**Prior biliary tract intervention**	*no*	64	42.2%	**0.003**	46.9%	0.406	4.8%	0.054	3.2%	0.059	4.7%	0.777
	*yes*	282	63.1%		53.5%		14.1%		11.5%		6.4%	
**Indwelling biliary tract drainage**	*no*	143	43.4%	**≤ 0.001**	51.7%	0.913	7.8%	**0.044**	7.1%	0.198	5.6%	0.823
	*yes*	205	69.8%		52.7%		15.4%		11.8%		6.3%	
**Stenosis**	*no*	161	50.9%	**0.006**	47.8%	0.133	8.2%	**0.033**	5.7%	**0.018**	5.0%	0.503
	*yes*	187	65.8%		56.1%		15.8%		13.4%		7.0%	
**Biliodigestive anastomosis**	*no*	248	59.5%	0.803	57.5%	**≤ 0.001**	11.5%	0.586	7.7%	**0.044**	5.6%	0.804
	*yes*	100	57.3%		37.1%		14.1%		15.3%		7.0%	
**Liver transplantation**	*no*	302	60.3%	0.201	56.6%	**≤ 0.001**	12.8%	0.628	9.7%	0.788	6.6%	0.333
	*yes*	46	50.0%		23.9%		8.7%		11.1%		2.2%	
**Multivariate analysis - independent predictors**												
		**OR (95% CI)**			***p*-value**							
***Enterococcus spp.***												
Indwelling biliary tract drainage		3.11 (1.94–5.00)			≤ 0.001							
**Enterobacterales**												
Bile collection > 48 h from admission		0.41 (0.25–0.66)			≤ 0.001							
ICU^a^		0.38 (0.23–0.63)			≤ 0.001							
**3GCRE**												
Male sex		2.61 (1.15–5.95)			0.022							
Charlson Index ≥5		1.98 (1.00–3.92)			0.049							
MDR carrier		3.57 (1.73–7.37)			≤ 0.001							
**MRGN**												
Male sex		4.27 (1.42–12.82)			0.010							
MDR carrier		2.47 (1.04–5.86)			0.040							
Percutaneous bile collection		2.61 (1.13–6.01)			0.024							
Stenosis		2.66 (1,02-6,95)			0.046							
***P. aeruginosa***												
ICU^a^		4.06 (1.59–10.34)			0.003							

MRGN, multidrug resistant gram negative bacteria as defined by KRINKO [6]

MDR, multi-drug resistant bacteria including MRSA, MRGN, and VRE

^a^ICU, defined as treatment on ICU before or during bile sampling

^b^Antibiotic pre-treatment within 3 months prior to admisson

**Fig. 2:**
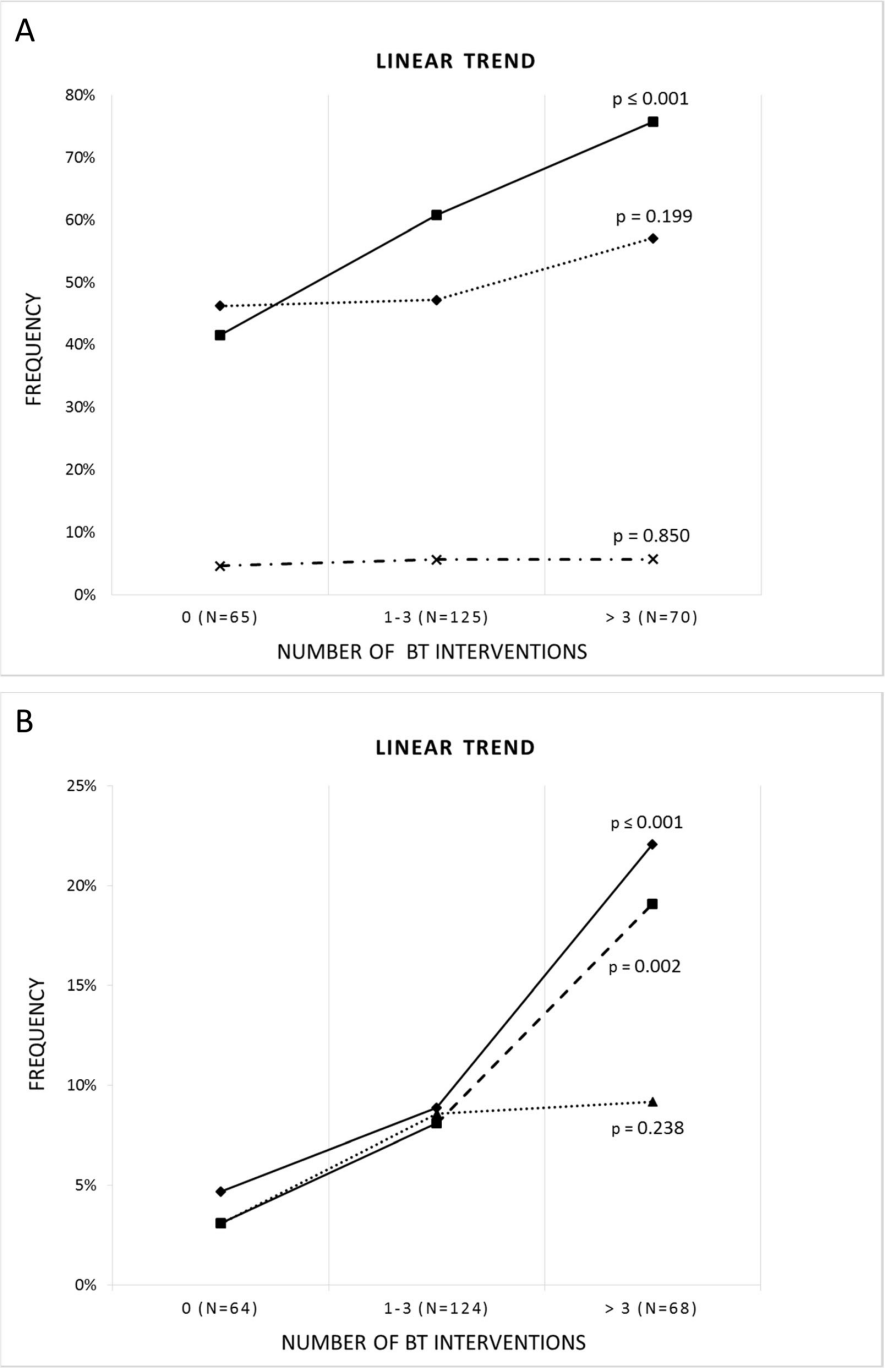
**a** Linear trends between the number of biliary tract (BT) interventions and the frequency of infections by enterobacterales (diamonds), enterococci (squares), and *P.aeruginosa* (crosses). **b** Linear trends between the number of BT interventions and the frequency of infections by third generation cephalosporine-resistant enterobacterales (3GCRE, diamonds), multi-resistant gram-negative rods (MRGN, squares), and vancomycin-resistant enterococci (VRE, triangles), respectively

After multivariate analysis, bile sampling later than 48 h after admission and ICU treatment before or whilst sampling remained negative predictors for isolation of enterobacterales from bile culture (OR 0.41 and 0.38, respectively), whereas the presence of a biliary drainage remained a strong risk factor for the isolation of enterococci (OR 3.1) (Table 4). In line with this, a significant linear trend was observed between the number of interventions and the frequency of enterococcal infections (*p* ≤ 0.001) amongst 260 patients in whom the exact numbers of previous biliary tract interventions could be determined (Fig. 2).

As to the association of patient factors with antibiotic resistance, multivariate analysis showed that male sex and MDR carrier status (comprising MRSA, MRGN, or VRE) were independently associated with an increased prevalence of both 3GCRE and MRGN in bile cultures (Table 4). In 41% of individuals with MRGN carrier status, MRGN were also cultured from bile sampled during acute cholangitis (compared to only 8% in individuals without, p ≤ 0.001). In addition, a high level of comorbidity was associated with an increase in 3GCRE, while percutaneous bile sampling and the presence of a biliary stenosis was associated with more MRGN (Table 4). By linear trend, patients with more than three biliary tract interventions were significantly more likely to present with 3GCRE or MRGN biliary tract infections (Fig. 2). Regarding VRE, 20% of known carriers also had VRE isolated from bile cultures during acute cholangitis (compared to 7% of patients without carrier status).

### Antibiotic therapy

In 229 patients the empirical antibiotic regimen could be determined from the clinical records. One hundred thirty-six patients (59%) received monotherapy, and 93 patients (41%) were treated with a combinational regimen. The most commonly prescribed substances were cephalosporines, followed by piperacillin/tazobactam and fluorochinolones (Table 5). The most frequent combinational therapy consisted of a beta-lactam plus metronidazole. With the regimens employed, bacterial organisms isolated from bile were covered in only 51% of patients (taking into account all isolates of a sample). Even more importantly, empiric coverage of blood culture isolates was only 69%. Amongst patients with bacteremia, three out of 11 (27%) patients with inadequate empirical therapy deceased, compared to only two out of 22 (8%) patients with adequate therapy.

**Table 5:** Empirical antibiotic treatment

	**Frequency**	
		*(n)*
**Mono vs. combinational therapy**		
Mono	59.4%	136
Combinational	40.6%	93
2 drugs	33.6%	77
≥ 3 drugs	7.0%	16
**Prescribed antibiotic substances**		
Cephalosporine^a^	25.8%	59
Piperazillin/tazobactam	23.6%	54
Fluorochinolon	21.4%	49
Carbapenem	18.8%	43
Metronidazole	18.8%	43
Ampicillin/sulbactam	16.2%	37
Vancomycin	9.2%	21
Other	14.8%	34

A total of 229 patients were studied

^a^2nd or 3rd generation

The primary goals of antibiotic therapy in ascending cholangitis are to prevent and treat systemic spread of the infection. In order to test whether carbapenem-sparing antibiotic therapy might be used at least for certain selected patients, we analysed antibiotic coverage in patient subgroups defined by simple patient-associated factors. For this analysis, the efficacy of the antibiotic therapy was evaluated in respect to the blood culture isolates, because a positive blood culture obviously represents proof of systemic spread. As shown in Table 6, blood culture isolates obtained from patients with community-acquired cholangitis (biliary sampling within 48 h of admission), without indwelling biliary drainage, and no ICU treatment would have been covered by piperacillin/tazobactam by 100%. In comparison, only a 78% coverage rate would have been achieved by third generation cephalosporines. In these patients, the use of carbapenems does not further improve antibiotic coverage.

**Table 6:** Patient-based coverage of blood culture pathogens

**Risc factor**			**CTX**		**TZP**		**CP**	
		**n**		***p*-value**		***p*-value**		***p*-value**
**Time of bile collection from admission**	≤ 48 h	21	71.4%	0.062	81.0%	0.044	95.2%	0.101
	> 48 h	23	43.5%		52.2%		78.3%	
**ICU**^**†**^	*no*	29	69.0%	0.024	75.9%	0.053	89.7%	0.376
	*yes*	15	33.3%		46.7%		80.0%	
**Indwelling biliary tract drainage**	*no*	22	68.2%	0.128	81.8%	0.026	90.9%	0.380
	*yes*	22	45.5%		50.0%		81.8%	
**Any of the above risk factors**	*no*	9	77.8%	0.155	100.0%	0.016	100.0%	0.181
	*yes*	35	51.4%		57.1%		82.9%	

Percentages indicate the proportion of patients for whom all blood culture pathogens would have been covered by the respective antibiotic

*CTX* cefotaxime, *TZP* piperacillin/tazobactam, *CP* carbapenem

^†^ICU, treatment on ICU before or during bile sampling

## Discussion

Empirical therapy of cholangitis represents an educated guess based on spectrum and likelihood of causative pathogens and their expected antimicrobial susceptibility. Accordingly, the current Tokyo guideline recommends third generation cephalosporines, piperacillin/tazobactam or carbapenems for empirical treatment the choice of which should be guided by local susceptibility data and the severity of the infection. For severe cases and hospital-acquired infections anti-pseudomonal agents and coverage of enterococci are recommended. Because optimal empirical treatment may vary greatly between different institutions, a multi-disciplinary approach is suggested [4].

Although these recommendations provide a useful framework in general, the selection of antibiotic therapy is still associated with various uncertainties. Without guidance by local susceptibility data, it is difficult for the attending physician to make his choice between the three betalactam-options. Carbapenems seem to be the safest choice, because of their bactericidal activity against most gram-negative rods resistant to third generation cephalosporins and acylureidopenicillins. However, increased carbapenem usage promotes carbapenem-resistance, which is regarded a major global public health problem. According to recognized principles of antibiotic stewardship, it is therefore widely advocated that carbapenems should be used in a restrictive manner in order to minimize the selection pressure favouring growth of carbapenem-resistant bacteria.

Even if local susceptibility rates are available, they are usually not provided in respect to specific disease entities despite considerable disease-specific variations making the choice of the optimal empirical therapy difficult. Owing to the rising prevalence of resistant gram-negative rods, carbapenems often seem to be the only effective option left. Particularly, empirical therapy guided by microbiological data only may fall short in respect to the goals of modern antibiotic therapy if patient-related response rates are not taken into account. Therefore, the present study aimed to analyse both microbiological as well as patient data in order to optimize empirical therapy of acute cholangitis. To this end, our analysis identified risk factors for 3GCRE and/or MRGN. Although we did find a high overall prevalence of cepholosporine resistant enterobacterales in bile and blood cultures of our patients, our results imply that piperacilline/tazobactam is a highly effective option for patients with community-acquired cholangitis, without pre-existing biliary prosthesis, and not treated on an ICU.

In our cohort, polymicrobial growth occurred in almost 70% of bile samples with enterobacterales and enterococci being the most common pathogens, each accounting for about one third of bile isolates. Anaerobes were detected in less than 10% of our patients. These results are consistent with other recent reports [7–10] and revise the classic view that both enterobacterales and anaerobes are the most important pathogens in biliary tract infections [11]. Bacteremia was detected in one third of cases with available blood culture results. Reflecting their differing virulence, and in agreement with a recent large multicenter trial on biliary infections, enterobacterales comprised almost 60% and enterococci only 20% of blood culture isolates [2]. In line with previous studies, our multivariate analysis found the presence of a biliary prosthesis an independent predictor for enterococcal isolation from bile [3, 9, 12, 13]. The Tokyo guideline recommends empirical treatment covering enterococci in severe community-acquired cholangitis – and in health-care associated infections if patients are colonized or enterococci are otherwise of concern. We agree on this but would like to point out that enterococcal coverage should particularly be considered in patients carrying an indwelling biliary drainage. As stated in the guideline, VRE carrier status should be taken into account in the choice of substance [4].

As to the frequency of resistant enterobacterales, we found an 80% susceptibility of bile isolates to fluorochinolones and an almost 90% susceptibility of blood isolates to third generation cephalosporines. Thus, on first view, all antibiotic choices recommended by the Tokyo guideline seem to be appropriate for our patients. However, when coverage was analysed in a patient-wise manner, a different picture emerged. Although the antibiotics prescribed largely reflected the Tokyo guideline recommendation, empirical therapy covered bile isolates in only 51% of patients. In patients with bacteremic infections, coverage was 69% which is still far less than desired. Probably owing to both the high proportion of polymicrobial growth and the high prevalence of resistant bacteria in bile and blood cultures, the actual antibiotic coverage was unacceptably low.

Epidemiological data from Germany suggest a frequency of 3GCRE of about 15% in hospitals [14]. In an analysis of cholangitis cases from two other German university hospitals, ESBL producing *E.coli* were detected at a rate of 31% [10]. In our cohort, the highest numbers of cholangitis associated with either 3GCRE or MRGN were found amongst patients carrying MDR pathogens. In those known to be colonized, MRGN were detected in 41% of bile cultures. Individuals with a high burden of comorbidity were also more prone to infections with 3GCRE. Furthermore, the presence of biliary stenosis was associated with an increased risk for MRGN, and we observed a linear trend between the proportion of patients infected with 3GCRE and MRGN and the number of prior biliary tract interventions. Thus, it seems reasonable to cover patients with carrier status, a high burden of comorbitiy or the presence of biliary stenosis and repeated biliary interventions (in our study > 3 interventions) with a carbapenem for empiric therapy. In addition to these risk factors, we found male sex to be an independent risk factor for isolation of 3GCRE or MRGN. In fact, a previous study on acute cholangitis from two German university centers also reported an association of male sex with infections caused by ESBL *E.coli* [10]. At the time being, the causal relationship for this association is largely unclear. Therefore, we would presently not suggest to cover all male cholangitis patients with a carbapenem.

Having defined predictors for infection with 3GCRE or MRGN, the question arises, whether patient subgroups can be identified that can still be effectively treated with a carbapenem-sparing therapy even in a tertiary referential center with a high prevalence of resistant pathogens. According to our data, piperacillin/tazobactam would represent a highly effective option for patients presenting with community acquired cholangitis, without indewelling biliary drainage, and not on an ICU. Of note, if only resistance data would have been considered (frequency of 3GCRE in blood culture isolates 11.4%), the choice would have been a third generation cephalosporine. However, if analysed in a patient-based manner, the coverage by cefotaxime was poor (< 80%) for unselected patients as well as for all subgroups investigated. The better results for piperacillin/tazobactam in our cohort are explained by the coverage of ampicillin-susceptible enterococci, piperacillin-susceptible *P.aeruginosa* and anaerobes but not 3GCRE that were tested susceptible to piperacillin/tazobactam in vitro. This is important because piperacillin/tazobactam was inferior to carbapenems in the treatment of blood stream infections caused by *E.coli* and *K.pneumoniae* resistant to third generation cephalosporines [15]. Aside from these considerations, our results demonstrate that i) efficacy of antibiotic regimens should be assessed in a patient-based manner, and ii) combined analysis of microbiological and patient data is a valuable tool for deliberate and efficacious use of antibiotics in settings with a high prevalence of resistance.

Since this paper focuses on antibiotic therapy, we have not included a risk analysis for biliary candidiasis. For completeness, a high Charlson Index, bile sampling later than 48 h after admission, and an indwelling drainage were independent risk factors for a fungibilia in our cohort. An indwelling drainage and/or ICU treatment were risk factors for candidaemia .

An important limitation of this retrospective study is the lack of documented information regarding administration of antibiotic therapies prior to microbiological sampling. Owing to its potential effect on microbial spectra and prevelance of resistancies, prior antibiotic exposure should be considered when selecting an empiric regimen. Still, the predictors for a carbapenem-sparing therapy identified in our study can be applied to patients without recent antibiotic therapy and to those in which information on antibiotic exposure cannot be reliably obtained from the patient’s history.

## Conclusion

In summary, we found a high prevalence of 3GCRE or even MRGN in bile and blood samples obtained from patients with acute cholangitis as would have been expected for a tertiary referential center with associated liver transplant unit. Carrier status, a high burden of comorbitiy, the presence of biliary stenosis, and repeated biliary tract interventions were identified as risk factors for isolation of resistant gram negative bile culture isolates. Only the combined analysis of microbiological and patient data revealed piperacillin/tazobactam as an excellent choice for empiric therapy of patients with community acquired cholangitis, no biliary prosthesis, and no previous or ongoing ICU treatment. The study thus provides an example of how the Tokyo guideline recommendations can be specified for a particular institution on the basis of local microbiology and individual patient characteristics.

## Data Availability

The datasets used and/or analysed during the current study are available from the corresponding author on reasonable request.

## Abbreviations

3GCRE: Enterobacterales resistant to third generation cephalosporines
CoNS: Coagulase-negative Staphylococci
ESBL: Extended spectrum beta-lactamase
KRINKO: Commission for Hospital Hygiene and Infection Prevention at the Robert Koch Institute
MDR: Multi-drug resistant
MRGN: Multi-resistant gram-negative rods
VRE: Vancomycin-resistant enterococci
